# Neurometabolic Stability and Heritability in the Adolescent Brain: A Preliminary Longitudinal Twin MRS Study

**DOI:** 10.1002/nbm.70292

**Published:** 2026-04-19

**Authors:** Christoph Hamann, Andjela Markovic, Julien Caccia, Andrzej Badek, Salome Wild, Kristina Adorjan, Michael Kaess, Ruth Tuura, Leila Tarokh

**Affiliations:** ^1^ Division of Child and Adolescent Psychiatry and Psychosomatic Medicine, Department of Pediatrics Inselspital, Bern University Hospital, University of Bern Bern Switzerland; ^2^ Translational Research Center, University Hospital of Psychiatry and Psychotherapy University of Bern Bern Switzerland; ^3^ Department of Psychology University of Fribourg Fribourg Switzerland; ^4^ University Hospital of Child and Adolescent Psychiatry and Psychotherapy University of Bern Bern Switzerland; ^5^ Graduate School for Health Sciences University of Bern Bern Switzerland; ^6^ MR‐Research Centre University Children's Hospital Zurich Zurich Switzerland; ^7^ University of Zurich Zurich Switzerland; ^8^ Children's Research Centre University Children's Hospital Zurich Zurich Switzerland

**Keywords:** development, early adolescence, heritability, MRS, neurometabolites

## Abstract

Early adolescence is a critical period of brain development. This study examined regional neurometabolite ratios, their longitudinal stability, and their heritability during early adolescence. Forty‐four adolescent twins (mean age: 13.25 years; SD = 1.01 years; 18 females; 25 monozygotic twins) underwent magnetic resonance spectroscopy (MRS) of the prefrontal cortex (GABA‐edited MEGA‐PRESS) and thalamus (short‐echo‐time PRESS) at baseline and 6 months later (*n* = 32; mean age: 13.72 years; SD = 0.97; 14 females; 19 monozygotic twins). We observed stable neurometabolite ratios in both regions across time. Moderate genetic contributions were estimated for Glx (glutamate and glutamine)/(Cr + PCr) in the prefrontal cortex as well as for glutamate/(Cr + PCr) and total choline‐containing compounds (PCho + GPC)/(Cr + PCr) in the thalamus. These preliminary findings highlight region‐specific and genetically influenced neurometabolic markers in early adolescence.

## Graphical abstract

In this preliminary longitudinal MRS study of early adolescent twins, neurometabolite ratios in the prefrontal cortex and thalamus appeared relatively stable over a 6‐month interval. Moderate heritability estimates were observed for prefrontal Glx (glutamine + glutamate) as well as thalamic glutamate and choline compounds, suggesting possible region‐specific genetic contributions.

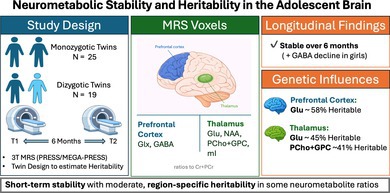

**Abbreviations**
AIC: Akaike information criterion
C: shared environmental factors
Cr: total creatine (Cr + PCr)
DLPFC: dorsolateral prefrontal cortex
DZ: dizygotic twins
E: unique environmental factors and measurement error
ET: echo time
GABA: gamma‐aminobutyric acid
Gln: glutamine
Glu: glutamate
Glx: glutamate and glutamine
GPC: glycerophosphorylcholine
*h* ^2^
: Falconer's heritabilit
ICC: intraclass correlation coefficient
mI: myo‐inositol
MRS: magnetic resonance sprectroscopy
MZ: monozygotic twins
NAA: *N*‐acetyl‐aspartate
PCho: phosphorylcholine
PRESS: Point RESolved
SEM: structural equation modeling
TR: repetition time

## 1 Introduction

Adolescence is a critical period of development marked by rapid cognitive and emotional maturation, driven by significant neural reorganization and brain maturation. Among the key factors influencing this process are neurotransmitters, the chemical messengers that regulate neural communication. Neurotransmitters play a fundamental role in shaping the brain's structural and functional maturation, influencing behavior, cognition, and emotional regulation during adolescence [1, 2]. Neurotransmitter systems are recognized as modulators of neurodevelopmental processes including synaptic pruning, critical period plasticity, and circuit maturation [3, 4]. However, the specific role of metabolites driving these processes remains less certain, and they are best considered as markers of underlying maturational changes rather than primary drivers of development. Two brain regions that undergo marked maturation during the adolescent years are the prefrontal cortex and limbic system. These regions are critical for decision‐making, impulse control, and emotional processing—functions that are especially dynamic during adolescence [5, 6]. Neurotransmitter systems, including dopaminergic, serotonergic, glutamatergic, and gamma‐aminobutyric acid (GABA)‐ergic pathways, are actively involved in modulating these processes [7, 8, 9]. Alterations in the developmental trajectory or functional balance of excitatory and inhibitory neurotransmitter systems have been linked to impaired circuit maturation and increased psychiatric vulnerability [10, 11, 12].

Understanding the role of neurotransmitters and neurotransmetabolites in adolescent brain development is essential for elucidating the biological basis of typical and atypical developmental trajectories. In this paper, we make use of magnetic resonance spectroscopy (MRS) to understand developmental changes in neurometabolic systems. MRS is a noninvasive imaging technique that enables the quantification of key neurometabolite ratios relative to total creatine (Cr + PCr), which is commonly used as an internal reference [13]. In this paper, we focus on the following neurometabolites as they have been shown to impact either mental health and/or developmental processes: glutamate (Glu), GABA, *N*‐acetyl‐aspartate (NAA), total choline‐containing compounds (phosphorylcholine and glycerophosphorylcholine [PCho + GPC]), myo‐inositol (mI), and glutamine together with glutamate (Glx). Glutamate is the brain's primary excitatory neurotransmitter, critical for synaptic plasticity, learning, and memory [14]. GABA, on the other hand, is the primary inhibitory neurotransmitter, essential for maintaining the balance of excitatory and inhibitory signaling in the brain [15]. The interplay between Glutamate and GABA is particularly important during development, as it shapes neuronal circuitry and functional connectivity [16, 17, 18]. NAA is a marker of neuronal health and function, often used to assess changes in neuronal integrity during development [19]. Total choline (Cho)–containing compounds (PCho + GPC) are involved in cell membrane synthesis and turnover, reflecting processes such as myelination and membrane remodeling, both of which are vital during brain maturation [20]. Myo‐inositol serves as a marker of glial activity and osmoregulation, providing insight into the nonneuronal components of brain development [21]. Finally, Glutamine is closely related to glutamatergic metabolism, offering additional information about neurotransmitter cycling and metabolic processes [22, 23, 24]. By investigating these specific metabolites, we aim to shed light on how neurometabolic systems evolve during critical periods of brain development and how these changes may underlie behavioral outcomes. MRS‐visible metabolites are often discussed in the context of neurotransmission, but it is important to note that the signal primarily reflects large intracellular metabolic pools rather than synaptic release. Nevertheless, functional MRS studies demonstrate that these measures can serve as proxy markers of excitatory and inhibitory neurotransmission under certain conditions [25, 26, 27, 28].

We examined the aforementioned neurometabolite ratios in the prefrontal cortex and the thalamus. The prefrontal cortex plays a central role in higher‐order cognitive functions, supporting top‐down executive control, working memory, attentional regulation, and the modulation of impulsive and emotional responses [29, 30], all of which undergo significant maturation during adolescence. The thalamus, on the other hand, functions as a relay hub, integrating and gating sensory and subcortical inputs relevant for affective, motivational, and cognitive processes, and is increasingly recognized as contributing to cognitive regulation during development [31, 32, 33, 34].

To explore the heritability of these neurometabolite systems, we leverage a twin study design, which provides a unique opportunity to disentangle genetic and environmental influences on brain development. By comparing metabolite ratios in monozygotic (identical) twins, who share nearly 100% of their genetic makeup, and dizygotic (fraternal) twins, who share approximately 50% of their genes, we can estimate the degree to which these systems are genetically determined. Twin studies allow us to investigate whether variability in neurometabolite systems is predominantly driven by genetic factors or shaped by environmental influences [35]. This approach is particularly valuable during adolescence, a period when both genetic predispositions and environmental factors interact to influence brain development and mental health [36].

## 2 Methods

### 2.1 Participants

Forty‐four adolescents (mean age: 13.25 years; SD: 1.01; 18 females; 25 monozygotic twins) were recruited through a twin study investigating early adolescent maturation [37, 38, 39, 40, 41]. Contact was established through flyers and a website. A medical doctor screened the participants for exclusion criteria, which included a personal history of chronic or current disease, preterm birth before the 30th gestation week, use of medication, loss of consciousness > 5 min, and mental or physical handicap. The local ethics commission approved the study, which was carried out according to the Declaration of Helsinki. All participants and their parents gave written informed consent to participate in the study and were reimbursed for their time and effort. Longitudinal data with a second MRS after 6 months (average time 192.97 days, SD: 17.28) was available for 32 of the 44 adolescents (mean age: 13.72 years; SD: 0.97; 14 females; 19 monozygotic twins). Nine participants were lost at follow‐up due to drop‐out and data from three participants had to be excluded because of clear fitting errors or other artefacts in the spectra, when crosschecked by experts (Details in Table 1).

**TABLE 1:** Demographic characteristics of the study sample at time 1 (T1) and time 2 (T2).

		T1	T2
Total study population	*n* (%)	44 (100)	32 (100)
	Mean age (years)	13.25	13.72
	SD (years)	1.01	0.97
	Female (%)	18 (41)	14 (43)
Monozygotic (MZ) twins	*n* (%)	25 (57)	18 (56)
	Mean age (years)	13.11	13.66
	SD (years)	1.08	1.08
	Female (%)	13 (30)	11 (34)
Dizygotic (DZ) twins	*n* (%)	19 (43)	14 (44)
	Mean age (years)	13.43	13.82
	SD (years)	0.9	0.84
	Female (%)	5 (11)	3 (9)

*Note:* The table summarizes sample size, age (mean and standard deviation), and sex distribution for the total study population and stratified by MZ and DZ twin status at baseline (T1) and follow‐up (T2). Values are reported as *n* (%) or mean (SD), as indicated.

Abbreviations: DZ, dizygotic; MZ, monozygotic; SD, standard deviation; T1, baseline; T2, follow‐up.

### 2.2 Magnetic Resonance Spectroscopy

**FIGURE 1:**
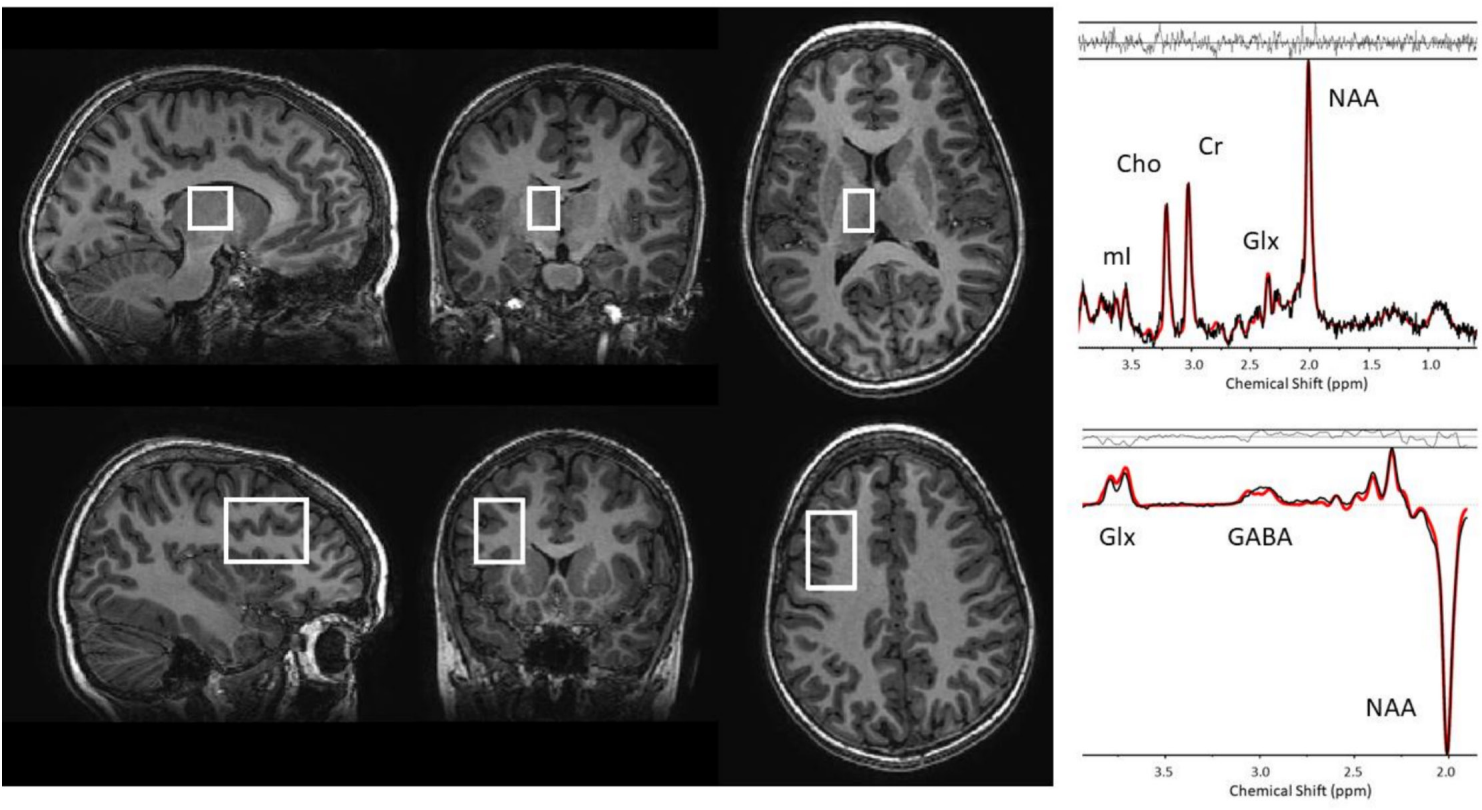
**Voxel positions and spectra** from a 12‐year‐old male participant. Single voxel PRESS and GABA‐edited MEGAPRESS spectra were acquired from voxels in the left thalamus (top panels) and left prefrontal cortex (bottom panels), respectively. Images are displayed in neurological orientation, with the left side of the image corresponding to the left side of the brain. The spectra depict the spectral data in black with the LCModel fit overlaid in red. The residuals are shown above each spectrum.

MRS scans were conducted in a 3T GE MR 750 scanner, using an eight‐channel receive‐only head coil. Single voxel Point RESolved (PRESS) ^1^H‐MR spectra were acquired from a 15 × 20 × 20 mm^3^ voxel of interest in the left thalamus using an echo time (TE) of 35 ms, a repetition time (TR) of 3 s, and 96 spectral averages. GABA‐edited MEGAPRESS ^1^H‐MR spectra were acquired from a 25 x 40 x 30‐mm^3^ voxel in the left prefrontal cortex, with a TE of 69 ms, a TR of 3 s, and 160 edit ON/OFF pairs (Figure 1). Editing pulses were applied at 1.9 and 7.5 ppm for the edit ON and OFF lines, respectively. Spectra were quantified using LCModel, a fully automated spectral fitting software package that estimates the metabolite ratio to total creatine (Cr + PCr) of each metabolite [42]. PRESS spectra were analysed using the standard GE basis set for TE = 35 ms, provided with LCModel, and MEGAPRESS spectra were analysed with a simulated basis set generated from density matrix simulations using published chemical shifts and coupling constants from Govindaraju et al. [43]. In LCModel, uncertainties are calculated as the percentages of the Cramer–Rao lower bound (CRLB) of the metabolite fit (% SD). The following metabolite ratios to total creatine (Cr + PCr) were analyzed: glutamate (Glu/(Cr + PCr)), total Cho‐containing compounds ((PCho + GPC)/(Cr + PCr)) comprised of phosphorylcholine (PCho) and glycerophosphorylcholine (GPC), myo‐inositol (mI/(Cr + PCr)), glutamate and glutamine (Glx/(Cr + PCr)), *N*‐acetyl‐aspartate (NAA/(Cr + PCr)), and gamma‐aminobutyric acid (GABA/(Cr + PCr)). Collected spectra underwent visual quality control for fitting errors and spectra where the relative CRLB for NAA or total creatine (Cr + PCr) exceeded 10% were excluded. MRS scans were conducted twice 6 months apart (+/− 4 weeks), with the same protocol administered at both time points. Example voxel positions and spectra are shown in Figure 1.

### 2.3 Heritability of Neurometabolites

**FIGURE 2:**
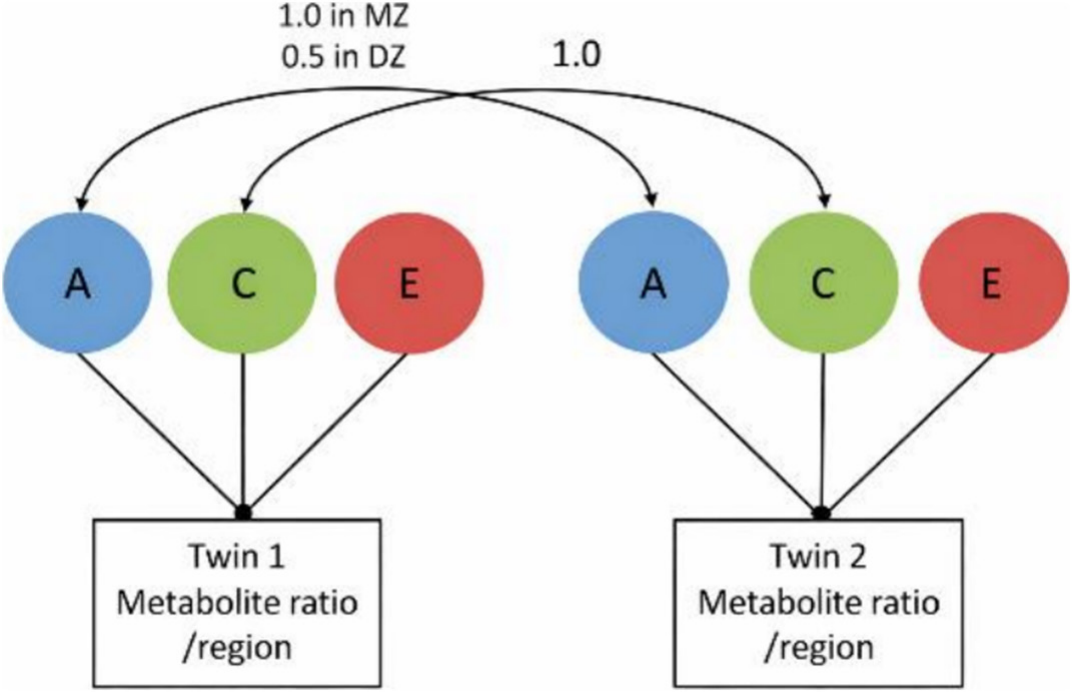
**Classical ACE twin model for metabolite ratios.** Schematic representation of an additive genetic (A), shared environmental (C), and unique environmental (E) model applied to metabolite ratios measured within a given brain region. For each twin, observed metabolite ratios are influenced by A, C, and E components. In this model, additive genetic influences are assumed to be correlated at 1.0 in monozygotic (MZ) twins and 0.5 in dizygotic (DZ) twins, whereas shared environmental influences are assumed to be correlated at 1.0 in both twin types. Unique environmental influences are uncorrelated between twins and include measurement error.

Structural equation modeling (SEM) with OpenMx in R (Boker et al., 2011) [44] was used to differentiate between genetic and environmental factors. SEM is a standard tool in twin research that allows the quantification of latent factors (e.g., genes and environment) based on observed data. The estimation of latent factors is achieved by the assumption that genetic concordance between monozygotic twins (MZ) is 1, whereas it is 0.5 in dizygotic twins (DZ). Conversely, both twin groups (MZ and DZ) share a familial and school environment and as a result, the shared environmental concordance is 1 for both sets. Unique environmental factors are elements, which are uncorrelated among both MZ and DZ twins and can include measurement error (Figure 2). Grounded on these postulations, structural equation modeling (SEM) can estimate the contribution of genetic factors (A), shared environmental factors (C), and unique environmental factors and measurement error (E). A, C, and E can vary between 0 and 1 and sum to 1, with 1 representing that 100% of the variance is explained by that factor. In some cases, assessments of C or A were close to zero and the full model (ACE) exhibited the highest Akaike information criterion (AIC), a measure of the goodness of fit of the model where lower values indicate a better fit. In these instances, the analysis was redone and the estimate that was close to zero (and nonsignificant) was excluded to achieve a better fit of the model [45, 46].

To be able to compare our results to other twin studies, we also conducted an intraclass correlation coefficient (ICC) analysis and calculated Falconer's heritability, *h* ^2^, defined as 2(rMz‐rDz). rMz and rDz are the correlation within the monozygotic and dizygotic twin pairs, respectively. These measures cannot differentiate between genetic and environmental influences, but rather are a measure of similarity, which we assume to be higher in MZ twins due to a higher proportion of shared genes among monozygotic as compared to dizygotic twins. Due to the small sample size at follow‐up we calculate heritability only on the baseline time point.

### 2.4 Longitudinal Changes in Neurometabolite Ratios

For statistical analyses, we used SPSS (Version 28.0.1.1; IBM Corporation, Armonk, NY, USA). To assess longitudinal changes in neurometabolite levels we used a repeated‐measures mixed‐model ANOVA, with age and sex as between‐subject factors and time as a within‐subject factor. All conclusions regarding change over time are based on this model. To aid interpretability we conducted a power analysis by converting the target effect size (Cohen's *d* = 0.59) into a minimal detectable difference in the original units using the observed standard deviation (Δ = *d* × SD). We additionally expressed Δ as a percentage of the sample mean (Δ% = Δ/mean × 100; Table 2).

**TABLE 2:** Neurometabolite ratios relative to Cr + PCr in the thalamus and the prefrontal cortex at time points one (T1) and two (T2).

		T1 (*n* = 44)	T2 (*n* = 32)	Δabs (Δ%)	ANOVA (time)	ANOVA (time × gender × age)	ANOVA (time × age sig)	ANOVA (time × gender)
Thalamus	Glutamate	1.372 (0.167)	1.392 (0.162)	0.098 (7.16)	0.318 (0.577)	1.206 (0.314)	0.486 (0.695)	0.328 (0.571)
	NAA	1.582 (0.138)	1.653 (0.207)	0.081 (5.14)	2.733 (0.109)	0.284 (0.755)	0.260 (0.854)	1.403 (0.246)
	PCho + GPC	1.679 (0.165)	1.7484 (0.193)	0.097 (5.78)	0.064 (0.802)	1.949 (0.161)	0.324 (0.808)	0.413 (0.526)
	mI	0.640 (0.152)	0.594 (0.098)	0.090 (13.98)	3.832 (0.060)	1.105 (0.345)	2.604 (0.072)	2.021 (0.166)
Prefrontal cortex	Glx	1.006 (0.108)	0.998 (0.096)	0.064 (6.35)	0.000 (0.994)	1.285 (0.298)	0.292 (0.831)	0.037 (0.850)
	GABA	0.315 (0.064)	0.301 (0.045)	0.038 (11.92)	0.154 (0.699)	0.095 (0.910)	2.227 (0.115)	**4.987 (0.037)**

*Note:* Means for each metabolite and standard deviation (SD) in parentheses are shown. In this table, Δabs represents the minimal detectable difference in absolute numbers and Δ% represents the minimal detectable difference in percent. Results of a mixed models ANOVA with between subject factors age and sex and within subjects factor time is reported for the following factors: time, time by age by gender interaction (time × gender × age), time by age interaction (time × age) and time by gender (time × gender) interaction. In these columns, *F*‐values are reported with *p* values in parentheses. Statistically significant values are in bold.

## 3 Results

### 3.1 Developmental Changes During Early Adolescence

To investigate developmental, age, and gender effects in MRS‐derived neurometabolite ratios, we conducted repeated measures mixed model ANOVA, with between‐subject factors of age and sex, and within‐subject factor of time (time 1 vs. time 2). Our analysis revealed no significant difference in neurometabolite ratios between baseline and follow‐up in the thalamus or in the left prefrontal voxel. We observed no age‐related effects for any neurometabolite ratio. Furthermore, no main effects for gender were found, however, we did find a significant interaction between time and gender for GABA ratios in the left prefrontal voxel, with a larger decline in girls than in boys (*F* = 4.987; *p* = 0.037). No other interactions (i.e., time*age*gender, time*age, time*gender) were found.

### 3.2 Heritability of Thalamic Neurometabolites

Concerning heritability in the thalamus (Table 3), ACE analysis estimated that the variability in glutamate (Glu/Cr + PCr) ratios was predicted by both genetic (45%) and unique environmental factors/measurement error (54%). Falconer's formula also indicated a possible strong genetic contribution, yielding a heritability estimate of 0.85 for glutamate (Glu/Cr + PCr). Consistently, ICCs were higher in monozygotic (MZ) twins (0.55) than in dizygotic (DZ) twins (0.06). Taken together, these findings suggest moderate heritability for glutamate (Glu/Cr + PCr) in the thalamus.

**TABLE 3:** Heritability of neurometabolite ratio relative to Cr + PCr.

		ICC (DZ)	ICC (MZ)	Pearson correlation (DZ)	Pearson correlation (MZ)	*h* ^2^	A	C	E	Best model
Thalamus	Glutamate	0.056 (0.470)	0.548 (0.126)	0.045 (0.915)	0.430 (0.214)	0.846	0.455	5.826E‐14	0.54	AE
	NAA	**0.742 (0.047)**	**0.706 (0.041)**	0.594 (0.121)	0.548 (0.101)	n.c.	1.10E‐13	0.591	0.41	CE
	PCho + GPC	0.65 (0.095)	**0.981 (0.0001)**	0.485 (0.224)	**0.980 (0.0001)**	0.99	0.419	0.509	0.07	CE
	mI	0.584 (0.814)	0.214 (0.363)	0.470 (0.287)	0.126 (0.729)	n.c.	1.82E‐14	1.604E‐15	1.00	CE
Prefrontal cortex	Glx	0.376 (0.658)	**0.815 (0.014)**	0.158 (0.708)	**0.731 (0.025)**	1.00	0.583	2.578E‐14	0.42	AE
	GABA	0.275 (0.353)	**0.718 (0.046)**	0.159 (0.733)	0.663 (0.052)	1.00	1.33E‐15	0.476	0.52	CE

*Note:* Intraclass correlation coefficients (ICC) for dizygotic (DZ) and monozygotic (MZ) twins with corresponding *p* values in parentheses. Pearson correlations *r* values (*p* values), Falconer's heritability (*h* ^2^), and results of the structural equation modeling (SEM) with A = genetic factors, C = shared environmental factors and E = unique factors and measurement error are shown and statistically significant values are in bold. When assessments of C or A were close to zero, the analysis was redone. If an estimate of A, C, or E approached zero, that factor was excluded to achieve a better fit of the model. The model with the best fit is reported in the last column. The abbreviation n.c. is when *h* ^2^ values were not calculated, because the correlation in DZ twins was larger than in MZ, resulting in negative values.

With regards to total Cho‐containing compounds, our data suggests moderate heritability. ACE analysis estimates that 41% of the variance in total Cho‐containing compounds is due to genetic factors. In line with this, ICC (ICC = 0.98) and correlation values (*r* = 0.98) for the MZ group were high and significant as compared to the DZ group (ICC = 0.65 and *r* = 0.46). Falconers' heritability (*h* ^2^) was 0.99. Based on ACE analysis, the influence of shared environmental factors is estimated at 50% plus a negligible influence of unique environmental factors (7%).

For NAA, a marker for neuronal integrity and density, we found similarly high ICC (MZ; ICC = 0.71 and DZ; ICC = 0.74) and correlation coefficients (MZ; *r* = 0.55 and DZ; *r* = 0.59) for both MZ and DZ twins. We interpret this to indicate high environmental influence in this parameter. This is supported by our ACE analysis, which estimated that shared environmental factors (59%) and unique environmental factors/measurement error (40%) account for much of the variance in this measure.

For mI, a glial marker, we found higher ICC values (ICC = 0.58) and higher correlation coefficients (*r* = 0.47) for DZ than for MZ (ICC = 0.21; *r* = 0.13) twins. This is supported by our ACE analysis, which estimated unique environmental factors/measurement error to be close to 100% with a negligible influence of genes and shared environmental factors.

### 3.3 Heritability of Neurometabolites in the Prefrontal Cortex

In the left prefrontal voxel, we find a genetic impact only on Glx, a mixture of both Glutamate and Glutamine (Glx/(Cr + PCr)). We find a high ICC (ICC = 0.82) and a significant Pearson correlation (*r* = 0.73) for the MZ as compared to DZ twins (ICC = 0.38; *r* = 0.16). This resulted in a Falconer's heritability estimate at the upper boundary (*h* ^2^ = 1); as boundary estimates can occur in modest samples, this value should be interpreted with caution. However, the ACE model suggests a genetic impact of 58%. The ACE model predicts the effect of unique environmental factors/measurement error with 41% for Glx. Taken together, these findings provide preliminary evidence for a genetic contribution to Glx/(Cr + PCr) ratios in the prefrontal cortex during early adolescence. In the left prefrontal voxel we were able to measure GABA, the brain's primary inhibitory neurotransmitter, due to the larger voxel and the use of spectral editing. We found larger ICC values for MZ (ICC = 0.72) compared to DZ (ICC = 0.27) twins. We find a correlation of 0.66, which does not reach significance (*p* = 0.052) for MZ twins and a correlation coefficient of 0.16 for DZ twins. Although Falconer's estimate reached the upper boundary (*h* ^2^ = 1), the ACE model indicated minimal genetic influence with higher estimates for shared environmental factors (47%) and unique environmental factors including measurement error (52%). Spectral quality criteria for both voxels are given in Table 4.

**TABLE 4:** MR spectral quality measures.

MR spectral quality measures		Median (range)
Thalamus	Linewidth (Hz)	5.5 (4.2–8.6)
	Glutamate (% CRLB)	7% (4%–13%)
	NAA (% CRLB)	3% (2%–9%)
	Total creatine (Cr + PCr) (% CRLB)	3% (2%–7%)
	PCho + GPC (% CRLB)	3% (2%–7%)
	mI (% CRLB)	6% (4%–15%)
Prefrontal cortex	Linewidth (Hz)	5.5 (3.7–12.8)
	GABA (% CRLB)	5% (2%–7%)
	Glx (% CRLB)	3% (2%–18%)
	NAA (% CRLB)	1% (1%–9%)

*Note:* Linewidth is measured in Hertz (Hz) and metabolite fit quality in percentage using the Cramér–Rao lower bound (% CRLB) for the thalamus and prefrontal cortex spectra. Reported are the median and the range for the metabolites of CRLB.

## 4 Discussion

This study investigated developmental changes in neurometabolite ratios during early adolescence and their heritability. Our preliminary findings indicate relative stability in neurometabolite ratios across time in both the prefrontal cortex and the thalamus, with notable genetic influences on glutamate and glutamine (Glx/(Cr + PCr)) in the prefrontal cortex as well as on glutamate (Glu/(Cr + PCr)) and total Cho‐containing compounds ((PCho + GPC)/(Cr + PCr)) in the thalamus.

### 4.1 Neurometabolite Stability and Developmental Trajectories

The observed stability of most neurometabolite ratios over 6 months in our sample in the thalamus and the left prefrontal cortex suggests a consistent neurometabolite milieu in early adolescence, despite significant neural maturation. This stability aligns with previous studies indicating limited fluctuation in neurometabolite ratios during adolescence in these regions [47]. Interestingly, when taking a more fine‐grained temporal perspective, neurometabolites as measured by MRS in the parietal lobe were found to be relatively stable overnight during late childhood and early adolescence [48]. In contrast, in young adults, an overnight change in neurometabolites, that is, a reduction in Glutamate and Glutamine, was described [49], suggesting that the overnight fluctuations in Glx may be age‐dependent. The contrasting findings between youth and adults may stem from the substantial interindividual variability in children and adolescents, which is influenced by ongoing maturational processes. In contrast to our findings, Perica et al. [47] and Shimizu et al. [50] as well as others [48] report a stable decline in Glu across the prefrontal cortex and other regions [51] throughout adolescence and young adulthood [52, 53]. However, the age range in these studies was much larger, and effect sizes are small to medium when reporting on adolescence. For example, Shimizu et al. report a small to medium effect size in the frontal cortex but compare data from two cohorts with age ranges from 4 to 11 versus 18 to 33 years of age [50]. Perica's data also indicate a small effect in Glu levels in the prefrontal cortex, based on a study of 144 participants aged 10–30 years [47]. Therefore, the developmental decline in Glutamate ratios to Cr + PCr in the prefrontal cortex might only arise when data from mid and late adolescence is also included.

In the left prefrontal cortex, GABA ratios show a sex‐specific decline, with a more pronounced reduction observed in females. This finding may reflect hormonal changes during puberty, which are known to influence GABAergic transmission [54]. For example, the prefrontal cortex is rich in estrogen receptors (ERα and ERβ), and estrogen can indirectly suppress GABA synthesis, possibly by modulating the expression of glutamate decarboxylase [55, 56]. Moreover, sex differences in synaptic pruning may also contribute, as some studies suggest that the prefrontal cortex undergoes earlier and more rapid pruning in females [57]. Despite this nuance, the overall stability of neurometabolite ratios suggests that the neurochemical milieu remains relatively unchanged during early adolescence. Although our analyses did not reveal significant changes in metabolite ratios across the follow‐up interval, these null findings should not be taken as definitive evidence of stability. With 32 participants completing both scans, the study was powered (90%) to detect medium effect sizes (*d* ≈0.59). Smaller effects may therefore have gone undetected, and larger samples are needed to determine whether more subtle developmental changes occur. Accordingly, our findings should be interpreted as indicative of short‐term stability within the limits of the study's statistical power, rather than as proof of the absence of change.

### 4.2 Heritability of Neurometabolite Ratios

Our heritability analyses revealed preliminary evidence for substantial genetic contributions to the Glx/(Cr + PCr) ratio in the left prefrontal cortex, with heritability estimates reaching up to 58%. Given the association of Glx‐related measures with glutamatergic systems [28], these findings suggest that variability in prefrontal Glx/(Cr + PCr) ratios may be partly influenced by genetic factors during adolescence, a period marked by ongoing maturation of executive and emotional regulation [58, 59]. Similarly, the thalamus showed moderate genetic influence for glutamate (Glu/Cr + PCr) and total Cho‐containing compounds ((PCho + GPC)/(Cr + PCr)), with shared and unique environmental factors accounting for a larger proportion of variance in other metabolites. Data on the genetic influence on neurotransmitter and neurometabolite levels during adolescence are scarce. However, a study in an adult Danish cohort by Legind et al. [60] reported heritability estimates of 16% for Glu, 33% for Glx, and 60% for Cho in the thalamus. While these estimates differ in magnitude and were obtained in an adult sample, they are consistent with a genetic contribution to thalamic neurometabolite ratios. Notably, the substantial heritability of total Cho‐containing compounds aligns with our observations in adolescents. Our findings are also broadly consistent with results from Hegarty et al. [61], who reported that reduced thalamic Glx ratios in twins with autism spectrum disorder showed substantial genetic contributions. While obtained in a clinical sample, these results further suggest that thalamic Glx‐related neurometabolite profiles may be influenced by genetic factors. We note that discrepancies in heritability estimates between structural equation modeling and ICCs for certain metabolites (e.g., GABA) highlight the complexity of genetic and environmental interactions during brain development and may also reflect our modest sample size. However, the sample represents a developmentally narrow age range during a period of heightened neurobiological plasticity, which is particularly relevant for studying dynamic neurometabolic changes [62, 63]. This high degree of age specificity helps reduce developmental heterogeneity and allows for more precise interpretation of neurochemical variation even within a smaller cohort. In addition, the observed inconsistencies may result from methodological differences between estimation approaches or from the influence of unmeasured environmental factors. Heritability estimates can vary depending on sample size, phenotypic measurement reliability and model specification [64]. Especially for neurochemical traits assessed via MRS, which are subject to both biological and technical variability, even modest deviations in measurement precision can differentially impact model‐derived estimates. Thus, while limited in size, our sample represents a developmentally well‐defined dataset that contributes to understanding early genetic and environmental influences on neurometabolite variability. Future studies with larger and more diverse cohorts will be essential to replicate and extend these findings.

### 4.3 Limitations and Future Directions

Our study's limitations include a relatively small sample size, particularly at follow‐up, which may reduce statistical power to detect longitudinal changes. Furthermore, our heritability analysis should be interpreted with caution given our modest sample size of 22 twin pairs (*n* = 44). While sufficient to detect large genetic effects, the study is underpowered to reliably quantify moderate to small genetic contributions. These findings should therefore be considered preliminary and warrant replication in larger samples. The narrow age range enhances developmental specificity, but limits generalizability across adolescence. Despite the fact that all twins were measured consecutively within an hour of each other, we did not collect all the MRS data during the same time of day, which could have an influence on the outcome [48, 65]. Another limitation concerns the relatively short follow‐up interval (~6 months), which captures only a narrow developmental window and may reduce the likelihood of observing robust maturational effects. Accordingly, the present findings should be interpreted primarily as evidence of short‐term stability rather than as definitive statements about developmental change. Additionally, we used metabolite/total creatine (Cr + PCr) ratios rather than absolute concentrations and assumed stability of total creatine (Cr + PCr) across adolescence and regions, which cannot be guaranteed. Future studies using water‐referencing are needed to confirm these effects. Nevertheless, establishing such short‐term stability during adolescence provides an important baseline for future studies, which will need to employ longer follow‐up periods and additional measurement points to more comprehensively characterize developmental trajectories. Finally, %CRLB thresholding can induce a bias towards higher metabolite ratios, particularly for metabolites with low concentration, as %CRLB values are inversely proportional to signal amplitude. In addition, spectral overlap in short TE spectra can result in strong correlations between detected metabolite signals, which can be partly masked in the presence of macromolecules and a background spectral baseline [66]. Therefore, metabolite ratios may not be statistically independent and can be intercorrelated in a complex way, such that metabolite ratios may reflect shared spectral variance rather than metabolite‐specific neurochemical effects. Future studies at high field strengths may help increase the spectral separation between metabolite signals, reducing spectral overlap and correlations between metabolites or with macromolecular signals. Incorporating measures of psychopathology [67], environmental factors such as stress [68] as well as social experiences [69] will provide a more comprehensive understanding of how the neurochemical milieu influences development. The importance of considering these factors when studying neurochemical trajectories is exemplified by a study done in adults with and without insomnia. This study showed that occipital GABA levels were higher in those with insomnia than in persons without disrupted sleep, while lower GABA levels were correlated with more time spent awake after sleep onset in both groups [65]. Incorporating sleep in future studies linking brain neurochemistry and mental health may be particularly fruitful, as disrupted sleep is commonly reported across mental health diagnoses [70, 71]. Moreover, multimodal‐imaging approaches combining MRS with functional and structural MRI could elucidate the interplay between neurochemical changes and brain connectivity in relation to brain maturation.

### 4.4 Conclusions

Our findings indicate the relative stability of neurometabolic ratios during early adolescence and provide preliminary evidence for genetic contributions to selected neurometabolite ratios in the prefrontal cortex and thalamus. While these results should be interpreted cautiously given methodological limitations, they add to the growing body of literature on adolescent brain development, emphasizing the need for continued longitudinal research to better characterize developmental trajectories and interindividual variability.

## Author Contributions

C.H. was a major contributor to writing the manuscript and was involved in data collection and data analysis. A.M. participated in data analysis, data interpretation, and manuscript editing. R.T. supervised MR data collection, contributed to data analysis and interpretation, and was a major contributor to writing and editing the manuscript. J.C. contributed to data analysis and manuscript writing. K.A., M.K., S.W., and A.B. were involved in manuscript editing. L.T. conceived the study, was a major contributor to writing the manuscript, and was involved in data collection and analysis. All authors read and approved the final manuscript.

## Funding

This project was funded by a grant from the Jacobs Foundation (to L.T.), with support from the Interfaculty Research Cooperation Grant “Decoding Sleep: From Neurons to Health and Mind” from the University of Bern (to L.T.) and the Swiss National Science Foundation (Grant 2003B_184943, to L.T.). The availability of the MEGAPRESS sequence was made possible through a research collaboration with GE Healthcare.

## Data Availability

The data that support the findings of this study are available on request from the corresponding author. The data are not publicly available due to privacy or ethical restrictions.
